# Prevalence of HIV, Hepatitis C and Hepatitis B Infection Among Detainees in a French Administrative Detention Centre

**DOI:** 10.1007/s44197-024-00238-0

**Published:** 2024-05-13

**Authors:** Sandrine Mancy, Pascale Fabbro-Peray, Sandrine Alonso, Hadi Berkaoui, Laetitia Lambremon, Hélène Vidal, Christophe Hilaire, Dorine Herrmann, Jennifer Dapoigny, Melanie Kinné

**Affiliations:** 1 Medical Unit of the Administrative Detention Centre (UMCRA), CHU Nîmes, Univ Montpellier, UMCRA 162 avenue Clément Ader, 30000 Nîmes, France; 2 Department of Biostatistics, Clinical Epidemiology, Public Health and Innovation in Methodology (BESPIM), CHU Nîmes, Univ Montpellier, Nîmes, France

**Keywords:** Human immunodeficiency virus, Hepatitis C virus, Hepatitis B virus, Migrants, Administrative detention centre, Rapid diagnostic test

## Abstract

**Background:**

In France, migrants constitute a significant proportion of people diagnosed with HIV, hepatitis C (HCV) and B (HBV). This study estimated the prevalence of these three viruses among detainees at a French administrative detention centre (CRA), through systematic Rapid Diagnostic Test (RDT) screening.

**Methods:**

This prospective, single-centre, cross-sectional, pilot study included detainees at the Nîmes CRA from February to December 2022. The primary endpoint was HIV, HCV and HBV prevalence determined by RDT. Secondary outcomes were: co-infections; study acceptability, reasons for non-inclusion, causes of non-contributory samples; and concordance between serological tests and RDT.

**Results:**

Among the 350 people agreeing to participate of 726 eligible, five refused the RDT, leaving 345 analysable participants for a participation rate of 47.5% (345/726). Participants were predominantly male (90%) with an average age of 31 years. The most common country of origin was Algeria (34%). Twenty (6%) had taken drugs intravenously and 240 (70%) had had unprotected sex within a median of 4.92 [1.08; 15] months. Virus prevalence was: 0% HIV; 4.64 [2.42; 6.86] % HCV; and 2.32 [1.01; 4.52] % HBV. Eleven (73%) of the RDT HCV positive cases were confirmed serologically. RDT detected one false-positive HCV case, as an anti-HCV Ac serological test was negative. Of the eight patients with positive HBV RDT, one declined the serology testing, thus 100% (7/7) of the tested RDT positive cases were confirmed by serology.

**Conclusion:**

The study highlighted the need to screen detainees for HIV, HCV and HBV infection and suitability of RDTs.

**Supplementary Information:**

The online version contains supplementary material available at 10.1007/s44197-024-00238-0.

## Introduction

The International Organization of Migration (IOM) defines a migrant as “a person who moves away from his or her place of usual residence, whether within a country or across an international border, temporarily or permanently, and for a variety of reasons” [1]. Migration is rising globally, and has increased substantially in Europe in recent years, with important implications for health services. In Europe, the prevalence of hepatitis B (HBV) and C (HCV) among migrants is estimated respectively at six times and twice that of the local population [2]. A systematic review showed that the prevalence of HIV infection was 2.25 times higher in migrants than in the local population in the USA and Europe [3]. In France, the prevalence of anti-HCV antibodies among migrants has been estimated at 1.6%, while the prevalence of chronic HBV has been estimated at 5.9% [4]. Despite the high prevalence of these three diseases in France, migrants face delays in diagnosis due to language barriers and their unfamiliarity with the French healthcare system [5].

In France, administrative detention centres (CRAs) are used to confine undocumented foreign nationals prior to repatriation to their country of origin. These facilities have been legalised in France since 1981 and are controlled by the border police. There are 25 CRAs in France, including four in the French overseas departments and territories [6]. People placed in a CRA are informed as soon as possible, in a language they understand, of their right to request a doctor visit [6–8]. Each CRA has a medical unit (UMCRA), usually attached to the local hospital. This unit is often the first contact between a foreigner and the French healthcare system [9]. Patients are systematically offered an appointment with a nurse for an initial assessment of their needs for prevention and somatic and psychological care [8].

However, the World Health Organization (WHO) recommends the development of a combined screening strategy for HIV, HCV and HBV in at-risk populations who are not or insufficiently screened (drug users, migrants, and homeless population) [10].There is little literature on healthcare management in UMCRAs in France, and no clear protocol for screening for these diseases in CRAs. As a result, centres have different practices and face financial, linguistic and medical obstacles [11].

The CRA in Nîmes does not currently systematically screen for HIV, HCV or HBV, and any screening performed is by serological tests. The main disadvantage of this method is the lag time waiting for results, as patients frequently leave the CRA before receiving their results. In 2022, 15.3% of detainees were released within 48 h of a decision by the judge at the Nîmes CRA [6]. RDT testing would allow all detainees to be screened for these three diseases immediately.

The aim of this study was to estimate the prevalence of HIV, HCV and HBV among detainees at the Nîmes CRA, by systematically offering RDT screening. The secondary objectives were to determine the prevalence of co-infections between HIV, HCV and HBV, to identify factors associated with these infection, to assess the feasibility of screening and to determine the concordance of the serological test in the event of a positive RDT.

## Methods

### Study Design

The TRODUMCRA study was a prospective, single-centre, cross-sectional, descriptive epidemiology, pilot study. The study was approved by the local ethics committee (comité de protection des personnes nord-ouest 1, #2021-A00544-37) and registered on clinicaltrials.gov (NCT05127187). This research was carried out in compliance with French law n°2012 − 300 of March 5, 2012 relating to research involving the human person, and with Good Clinical Practice and the Declaration of Helsinki.

### Settings and Participants

The study was proposed to detainees arriving at the Nîmes CRA from Monday to Friday, and those arriving on weekends or public holidays who were still present the following working day, due to the absence of a doctor on site on these days. Participants comprised detainees classed as illegal immigrants in France [7]. All adults detained at the Nîmes CRA were eligible for inclusion and there were no exclusion criteria. Inclusion lasted from February 7, 2022 to December 7, 2022 and participants were not followed-up. This study focuses on the prevalence of these diseases. Patients with a positive RDT were followed by UMCRA medical staff or referred to appropriate structure outside.

Patient data were collected by the physician at inclusion using a questionnaire (Supplementary Table 1): country of origin; language spoken; year of arrival in France; consultation with a doctor since arrival in France; previous screening for HIV, HCV and HBV infection; transfusion history; intravenous or snorting drug use; sharing or not of drug paraphernalia; unprotected sexual relations; and the presence of piercings or tattoos.

All detainees arriving at the Nîmes CRA underwent a standard interview by UMCRA staff. During this interview, a nurse informed the patient of the study, with the help of an interpreting service if necessary. The information letter was available in 17 languages (Supplementary Information 4). All participants provided oral informed consent.

Participant data were collected on a standardised electronic case report form (e-CRF) using (REDCap).

### Rapid Diagnostic Testing

Testing can be carried out using point of care Rapid Diagnostic Tests (RDTs) for rapid results. RDTs use finger prick blood sample or a saliva sample and provide a result within 30 min. The RDT screens for either antibodies (e.g. HIV and HCV) or antigens (e.g. HBV). These tests are complementary screening tools to serology, which is the reference method. Consequently, positive RDT result for HIV must be confirmed by a 4th generation ELISA test for HIV on a venous sample. Positive HBV RDTs must be confirmed by a blood test for HBsAg, anti-HBs antibody and anti-HBc antibody, and for HCV by a 3rd generation ELISA test for HCV followed by a viral load test (quantification of HCV RNA) on the same sample [10, 12]. A positive HBsAg test result means acute or chronic infection. A positive anti-HBs antibody test result indicates either a response to the hepatitis B vaccine or recovery from an acute hepatitis B infection. This result (along with a negative HBsAg result) indicates immunity against hepatitis B infection. The anti-HBc antibody is part of the virus, thus does not provide protection. A positive anti-HBc antibody test result indicates a past or present infection [13].

Immediate screening by RDT for HIV, HCV and HBV was carried out on finger prick samples taken by the nurse, according to the French Health Service (HAS) recommendations: [14]

- For HIV: RDT Nephrotek Toyo Insti ® - Sensitivity = 99.5%, Specificity = 99.3% [15].
- For HCV: RDT Nephrotek Toyo Insti ® Sensitivity = 99.5%, Specificity = 98.5% [16].
- For HBV: RDT Biosynex exacto first response ® Sensitivity = 98.98%, Specificity = 100% [14].

If one of the RDTs was positive, a serological test was performed during the same consultation, or the following day depending on time constraints. Serological tests were analysed in the microbiology and hospital hygiene department of Nîmes University Hospital (HBsAg, anti-HBs antibody, anti-HBc antibody, anti-VIH1 and anti-VIH2 antibody) by Alinity machine Abott ® and HCV RNA by Pathern Hologic ®.

### Outcomes

The primary endpoint was the prevalence of HIV, HCV and HBV determined by RDT. The secondary endpoints were: co-infection between any of the three viruses; analysis of risk factors by questionnaire; study acceptability rate, reasons for non-inclusion and causes of non-contributory samples; concordance between serological test results and RDT results, with prevalence recalculated if necessary.

### Bias

Selection bias was minimised by including subjects consecutively outside weekends. Subjects presenting to the CRA on weekends are not expected to differ from those arriving on weekdays. The information letter was translated into 17 languages, enabling inclusion of a greater number of patients. Classification bias was minimised by using RDTs with high sensitivity and specificity.

### Sample size

Sample size was calculated based on the expected prevalences of the viruses according to the endemic classification of the expected countries of origin. In 2021, the majority of undocumented migrants in France were from Eastern Europe, North Africa and sub-Saharan Africa [6]. HIV infection has a prevalence of around 3.2% in sub-Saharan Africa, which is classed as a highly endemic region [17]. North Africa and sub-Saharan Africa are in the medium endemic zone for HCV, with a prevalence of anti-HCV antibodies of between 2.5% and 5%. Eastern Europe is a low-endemic zone, with a prevalence of less than 2.5% [18]. HBV infection has a prevalence of over 8% in sub-Saharan Africa. North Africa has a prevalence of between 2% and 4%, while Eastern Europe has a prevalence of < 1% [19].

To show an HCV prevalence of 2% with a precision of ± 1.5% [18] and a bilateral alpha risk of 5%, 335 subjects needed to be included. This number made it possible to show a prevalence of HIV infection of 4% [17] with a precision of ± 2% and a prevalence of HBV of 8% [18] with a precision of ± 2.9%.To compensate for 5% potential problems of interpretation of test results, it was planned to include 350 subjects.

### Statistical Methods

Statistical results are presented as means and standard deviations for quantitative variables with a Gaussian distribution, and medians and quartiles for the other variables. For qualitative variables, the numbers and associated percentages are presented. Infection prevalences were estimated with their 95% confidence intervals, using the binomial distribution if the conditions were met, and the exact distribution otherwise. The prevalence of co-infections was estimated globally and according to the type of co-infection.

The feasibility of the study was assessed by calculating the rate of contributing subjects for prevalence calculations among the source population = number of contributing subjects for each pathology studied / number of subjects received at the CRA. The acceptance rate was calculated as the number of subjects with samples taken / number of subjects offered the study. The concordance of the serological test in the case of a positive RDT and the analysis of the prevalence of HIV infection, HCV and HBV was evaluated by the number of false and true positives.

Given the small numbers of subjects expected to carry the infection, multivariate analysis was not planned for the study of associated factors. A difference was considered statistically significant when the significance level of the test was less than or equal to 0.05. Statistical analysis was carried out by BESPIM, CHU de Nîmes, using SAS (SAS Institute, Cary, NC, USA) version 9.4.

## Results

### Participants

**Fig. 1:**
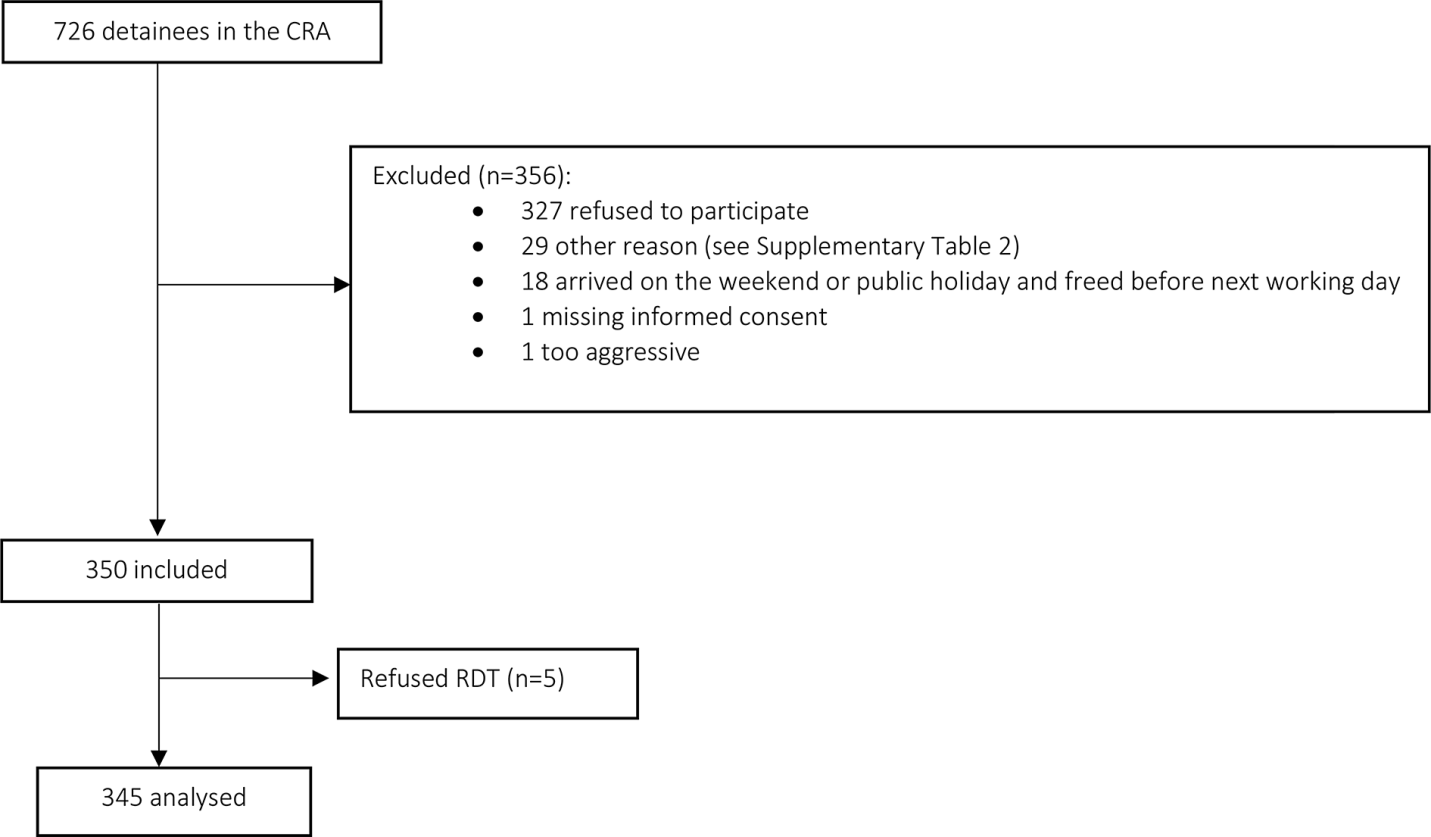
Patient flowchart

During the inclusion period, 726 detainees were eligible for inclusion. Of these, 20 (2.8%) were not offered the chance to enrol in the study, mostly due to arriving on a weekend and 327 (45%) refused to participate (Fig. 1). Of the 350 people agreeing to participate, five then declined to undergo the RDT, leaving 345 who accepted the RDT test. The included participants were predominantly male (*n* = 309, 90%) with an average age of 31 ± 8.9 years. The most common country of origin was Algeria (34%). The median time since arrival in France was 3 [1; 5] years. Most participants (*n* = 225, 65%) had already seen a doctor since arriving in France, and their last medical contact was a median 2.88 [1.2; 9.84] months ago. Non-French-speakers most commonly spoke English (26%), Arabic (23%) and Georgian (20%). Those having received a blood transfusion had each received it in a different country (Algeria, Bosnia-Herzegovina, Dominican Republic, France, Georgia, Nigeria and Tunisia). Patient characteristics are presented in Table 1.

**Table 1:** Patient characteristics. Data present as number (percentage) or median (1^st^ quartile; 3^rd^ quartile]

Characteristics	Population (*n* = 345)
***Sex***, male	309 (90%)
***Age***, years	31.03 ± 8.9 (18–63)
Country of origin*	
Algeria	119 (34%)
Tunisia	43 (12%)
Morocco	37 (11%)
Georgia	23 (7%)
Guinea	15 (4%)
Romania	14 (4%)
Nigeria	9 (3%)
Albania	8 (2%)
Bosnia-Herzegovina	6 (2%)
Turkey	6 (2%)
Other*	65 (19%)
***French-speakers***	276 (80%)
***Marital status***	
Single	163 (47%)
Married	83 (24%)
Couple	75 (22%)
Separated	24 (7%)
***Previous HIV, HCV and HBV screening***	153 (44%)
Last screening, years	1.02 [0.44; 2.15]
Positive HIV	0
Positive HCV	8 (5%)
HCV recovery	4 (3%)
Positive HBV	1 (1%)
***Blood Transfusion***	7 (2%)
***Drugs intravenously***	20 (6%)
***Drugs nasally***	118 (34%)
Shared equipment of drug users intravenously or nasally	57
***Unprotected sex***	240 (70%)
Within a median, months	4.92 [1.08;15]
***Piercing***	61 (18%)
By non-professional	2
***Tattoo***	104 (30%)
By non-professional	63

*for origins of < 2%, see Supplementary Table 3

### Prevalence

**Fig. 2:**
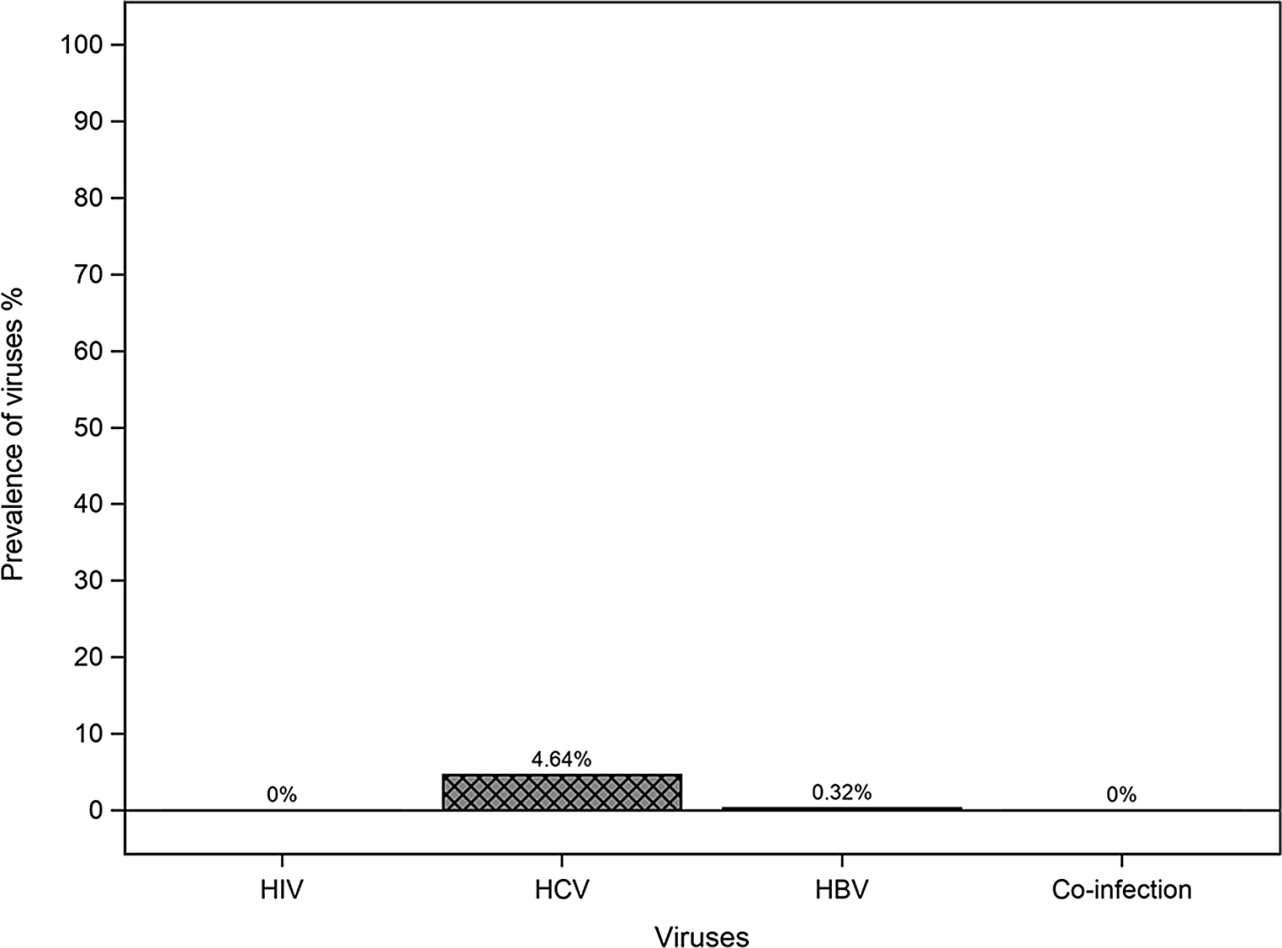
Virus prevalence in screened participants

All 345 individuals (100%) had a negative HIV RDT result. Sixteen participants were positive for HCV by RDT, hence a prevalence of 4.64 (95%CI: [2.42; 6.86])%. Eight participants were positive for HBV by RDT, with a prevalence of 2.32 (95%CI: [1.01; 4.52])%. None of the participants had a co-infection (Fig. 2).

Factors associated with a positive RDT for HCV included older age: RDT positive patients had a median age of 41 [29; 46] compared against 29 [25; 35] for negative RDT (*p* = 0.0065). Another factor was country of origin: *p* < 0.001: 8 (50%) Georgia, 2 (13%) Serbia, 2 (13%) Slovakia, 2 (13%) Bosnia-Herzegovina, 1 (6%) Armenia, 1 (6%) Tunisia for RDT positive patients (Table 2). Patients were more likely to be positive if they had ever had a screening test: 81% HCV-RDT positive individuals already had a screening test (*p* = 0.0023). As expected, participants already known to be positive were also more likely to return positive results: 50% of RDT positive individuals were already aware of their positivity. The proportion of intravenous drug users was significantly higher in the HCV-RTD positive group (63%) than in the HCV-RTD negative group (3%) (*p* < 0.0001), as were those with a tattoo: 9 (56%) versus 95 (29%) (*p* = 0.0264) (Table 2).

**Table 2:** Patient characteristics according to hepatitis positivity. Data presented as number (percentage) or median [1st quartile; 3rd quartile]

Characteristics	RDT Hepatitis C			RDT Hepatitis B		
	Positive *N* = 16	Negative *N* = 329	*p*-value	Positive *N* = 8	Negative *N* = 337	*p*-value
***Sex***, male	15 (94%)	294 (89%)	1	7 (88%)	302 (90%)	0.5898
***Age (years)***	41 [29; 46]	29 [25; 35]	0.0065	30 [27; 42]	29 [25; 36]	0.5722
Country of origin*			< 0.0001			0.0012
Armenia	1 (6%)	2 (1%)		0	3 (1%)	
Bosnia-Herzegovina	2 (13%)	4 (1%)		1 (13%)	5 (1%)	
Georgia	8 (50%)	15 (5%)		0	23 (7%)	
Guinea	0	15 (5%)		3 (38%)	12 (4%)	
Republic of the Congo	0	2 (1%)		1 (13%)	1 (0%)	
Senegal	0	4 (1%)		2 (25%)	2 (1%)	
Serbia	2 (13%)	3 (1%)		1 (13%)	4 (1%)	
Slovakia	2 (13%)	0		0	2 (1%)	
Tunisia	1 (6%)	42 (13%)		0	43 (13%)	
***Marital status***			0.3327			0.5256
Single	9 (56%)	154 (47%)		3 (38%)	160 (47%)	
Married	4 (25%)	79 (24%)		3 (38%)	80 (24%)	
Couple	1 (6%)	74 (22%)		1 (13%)	74 (22%)	
Separated	2 (13%)	22 (7%)		1 (13%)	23 (7%)	
***Arrival in France (years)***	3 [1.5; 8]	3 [1; 5]	0.3765	1 [0.5; 3.5]	3 [1; 5]	0.1305
***Medical consultation in France***	12 (75%)	213 (65%)	0.4002	4 (50%)	221 (66%)	0.4564
***Previous HIV, HCV and HBV screening***	13 (81%)	140 (43%)	0.0023	4 (50%)	149 (44%)	1
***Known HIV, HCV or HBV infection***	8	1	NA	0	9	NA
HCV	8	0		0	8	
HBV	0	1		0	1	1
***Transfusion***	1 (6%)	6 (2%)	0.2850	0	7 (2%)	1
***Injected drugs***	10 (63%)	10 (3%)	< 0.0001	0	20 (6%)	1
***Snorted drugs***	9 (56%)	109 (33%)	0.0570	0	118 (35%)	0.0548
***If snorted/injected drugs, shared materials?***	7	50	NA	0	57	NA
**Unprotected sex**	12 (75%)	228 (69%)	0.7843	7 (88%)	233 (69%)	0.4433
**Piercing**	2 (13%)	59 (18%)	0.7472	0	61 (18%)	0.3594
By non-professional	2	27	NA	0	29	NA
**Tattoo**	9 (56%)	95 (29%)	0.0264	2 (25%)	102 (30%)	1
By non-professional	7	56	NA	1	62	NA

*for origins, only countries with positive cases were shown

For HBV, only country of origin was associated with a positive RDT (*p* = 0.0012): 3 (38%) Guinea, 2 (25%) Senegal, 1 (13%) Serbia, 1 (13%) Democratic Republic of Congo, 1 (13%) Bosnia-Herzegovina for RDT positive patients.

### Study Feasibility

The rate of participation was 47.5% (345/726) and the acceptance rate was 48.9% (345/706) (Fig. 1).

The main reason for non-participation was patient refusal (*n* = 327, 86%), followed by constraints for 47 patients (12%) (Fig. 1). Twenty-five (7%) patients were released or transferred during their reflection period. Two (1%) refused to come to UMCRA, and 5 (1%) were initially included but refused to undergo the RDT. The two “other” reasons for refusal included a recent HIV-positive serology test and a recent serology test.

### Concordance

#### Hepatitis C

Sixteen RDTs were positive for HCV. However, four patients did not have the serology test: 1 declined; 1 had no venous access; and 2 left the CRA before the test. Of the 12 RDT positive cases tested, 11 (92%) were confirmed by the serological test. One HCV false-positive case was detected by RDT, as an anti-HCV antibody serological test was negative. Six of the samples had undetectable viral RNA.

#### Hepatitis B

Of the eight patients with positive HBV RDT, one did not consent to the serology testing, thus 100% (7/7) of the tested RDT positive cases were confirmed by the serological test. Six were negative for anti-HBs antibodies whereas one patient was positive.

## Discussion

The aim of the TRODUMCRA study was to estimate the prevalence of HIV, HCV and HBV among detainees at the Nîmes CRA, through systematic RDT screening. We found prevalence data for detainees of 0% for HIV, 4.64 [2.42; 6.86] % for HCV and 2.32 [1.01; 4.52] % for HBV.

Our study is the first to analyse the prevalence of HIV, HCV and HBV infection in a CRA. Screening was systematically offered to all detainees arriving at the Nîmes CRA during their first medical interview. Most studies on CRAs provide an overview of the practices of the various CRAs in mainland France, without a focus on disease screening methodology. The RDTs give a reliable result within 30 min of sampling. The doctor can inform the person of their results, promote prevention, and direct the patient towards organisations providing free information and screening and diagnosis centres, so that they can continue treatment should they be released quickly. The patient information sheet was translated into 17 languages, and an interpreting service was contacted if necessary, leading to inclusion of 20% of non-French-speaking patients. The majority of non-French speakers were able to read the information letter in their native language (Arabic (23%), English (26%) and Georgian (20%)). Only four patients spoke none of the 17 languages of the letter; the missing translations were Polish, Czech and Turkish. For these four patients, we used the interpreting service.

Few studies have been performed in administrative detention centres. In 2005, a national survey demonstrated the lack of treatment for viral hepatitis and HIV infection among detainees, despite being classed as a high-risk population [20]. In 2022, a circular relating to the UMCRA health system was rewritten by the French Ministries of the Interior and Solidarity and Health. However, infectious disease screening practices were not better described, contrary to the expectations of healthcare professionals [8]. In a study interviewing 27 healthcare professionals from six European countries (Greece, Italy, Croatia, Slovenia, Austria and Sweden) working in centres for refugees and asylum seekers, workers confirmed the lack of standardisation between countries on screening for infectious diseases [21]. They further noted that health-related information was not transferred in a standardised way between facilities within a single country. The lack of standardisation of screening and health assessments within countries, and the lack of harmonisation of these approaches between countries, further contributed to the perception among interviewees that service provision was haphazard [21]. To our knowledge, there are no studies analysing the prevalence of these infections in CRAs.

The prevalence of HCV in the TRODUMCRA study is higher than that found in the general population in metropolitan France and in the literature among migrants. In 2011, the prevalence of HCV in mainland France was 0.75% (95% CI: [0.62; 0.92]) [22]. A study of irregular migrants and refugees living in Naples and Caserta in Italy found a similar 4% prevalence of HCV. In our study, the countries of origin of the detainees were similar, although the proportion of detainees from North Africa was higher than in this study [23].. In the Netherlands, the prevalence of HCV among migrants is 0.99% (95% CI: [0.27; 3.52%]) in Gelderland and 1.17% (95% CI: [0.40; 3.39%]) [24]. A study of African migrants in Sicily found a HCV prevalence of 0.9% [25]. A French study of irregular migrants found a prevalence of HCV of 0.8% [26]. The STRADA study examined the prevalence of HIV infection and HBC and HCV among regular migrants seen at the French Office of Immigration and Integration. The prevalence of HCV was found to be 0.27% [27]. In our study, we found that country of origin, particularly Eastern European countries was associated with HCV prevalence. In 2019, HCV and HBV rates remained high in Europe. In Eastern Europe, the prevalence of HCV was 0.01%, compared with 0.005% in Western and Central Europe. This rate had remained constant from 2010 to 2019. In 2010, the age-adjusted prevalence rate of cirrhosis due to HCV was higher in Eastern Europe than in Central and Western Europe, with 2026.94 (95% CI: [1635.00; 2497.29]) cases per 100,000 people. In 2019, this prevalence rate remained stable in Eastern Europe, while a significant decrease was seen in Western Europe [28]. Intravenous drug use was associated with a higher prevalence of HCV in our study. A review of the literature showed a high prevalence of HCV among intravenous drug users ranging from 0.28 to 92.1%, median 65.6%, mainly from Kazakhstan, Georgia and Armenia [29]. The factors strongly associated with HCV are injecting drug use at least once in life and birth in a high-prevalence country [30]. The migrants who had received blood transfusions had a higher probability of having a positive test for HCV (95% CI: [1.91; 9.62]) in the STRADA study [27]. However, we found no association between a history of blood transfusion and a positive HCV test.

The prevalence of HBV in the TRODUMCRA study is higher than in the general population of mainland France, yet lower than the prevalence in the literature. In 2004, the prevalence of HBV in metropolitan France was 0.65% (95% CI: [0.45; 0.93]) [18]. The prevalence of HBV was 9% in a study of irregular migrants and refugees living in Naples and Caserta, Italy, and in a study of African migrants in Sicily [23, 25]. A French study of irregular migrants found a HBV prevalence of 3.1% [26]. In France, a study of migrants consulting a sexual health screening centre in Paris showed a prevalence of 5.5% (95% CI: [4.1; 7.3]) [31]. Populations in these two studies came from the same continents, mainly North Africa and sub-Saharan Africa, as our study. Indeed, country of origin (sub-Saharan Africa and Eastern Europe) was found to be significantly associated with an increased risk of HBV infection. This risk was underlined in a Finnish study with a similar prevalence of HBV to our study of 2.3% (95% CI: [1.5; 3.5%]), mainly among migrants from Somalia, a sub-Saharan African countries [32]. Originating from a country of high or medium prevalence (sub-Saharan Africa) was a factor associated with a positive HBV test in the French STRADA study [27]. In 2010, the age-standardised incidence rates of acute HBV were similar in the three European regions (Eastern, Central, and Western). From 2010 to 2019, similar decreases in incidence rates were estimated for Central Europe (–22.42%, 95% CI: [–35.82; − 5.40]) and Western Europe (–18.24%, 95% CI: [–31.41; − 3.38]), whereas Eastern Europe showed a larger decrease (–40.01%, 95% CI: [–52.24; − 24.82]). These decreases were consistent with trends in acute HBV incidence and prevalence rates. HBV prevalence was 0.028% in 2010 and 0.025% in 2019 in Western Europe [28].

No cases of HIV infection were observed in our study. The countries with the highest HIV prevalence are South Africa, Eswatini, Lesotho, Botswana and Zimbabwe [17], and none of the participants in our study came from these countries, possibly explaining the zero prevalence of HIV infection. One detainee did not wish to take part in the TRODUMCRA study because he had recently tested positive for HIV. Studies carried out in the Netherlands and Finland on migrants have similarly found no positive serology for HIV infection [24, 32].

The prevalences of HIV, HCV and HBV infection found in these various studies thus differ from those found in our study. This may be explained by the populations analysed in these studies, which are very different from the population in our study. In the literature, studies have focused either on legal migrants, authorised to enter and reside in the country in accordance with its legislation, or on undocumented migrants living in the community.

Over half (56%) of the detainees in our study had never been tested for HIV, HCV or HBV infection. However, the WHO recommends a screening strategy for migrants. The UMCRA would appear to be an appropriate place to offer screening. Serology confirmed 15 positive HCV RDTs and seven positive HBV RDTs. One false-positive HCV RDT was identified, confirming that the RDT is a good means of screening.

Around half (48.87%) of the detainees at the Nîmes CRA agreed to be screened for these three diseases. One detainee refused to participate because the study took place during the Ramadan period. The time of year when the study was proposed may influence whether or not detainees took part. An in-depth evaluation for the reasons for refusal to take part was not planned, and it would be interesting to understand why half the detainees at the Nîmes CRA refused screening.

Our study analysed the prevalence of these diseases in a CRA in mainland France. Detainees in France’s CRAs are mainly of Algerian (23.5%), Albanian (11.6%), Moroccan (8.9%), Tunisian (8.2%) and Afghan (5.1%) nationalities. In Nîmes in 2021, 2.8% of detainees were Albanian, 2% Afghan and 34.4% Algerian [6]. However, the prevalence of HCV and HBV among Albanian refugees is high, at 70.6% (67.8–73.4%) and 22.2% (19.7–24.7%) respectively [33]. In our study, the prevalence of Albanian subjects was 2%, and no Afghans were included. In Afghanistan, the prevalence of HCV is estimated at 0.7% (95% CI: [0.5; 0.9%]) among the general population, and that of HBV is 1.9% [34, 35]. It is therefore likely, given the discordance of nationalities in the CRAs and the high prevalence of HCV and HBV, particularly among Albanians, that the population of the Nîmes CRA is not representative of the population of the various CRAs in France. A multicentre study of the prevalence of HIV infection, HCV and HBV would help determine whether screening needs are similar between CRAs in France. A clear methodological guide to infectious disease screening in CRAs would facilitate the management of detainees by healthcare professionals.

A limitation of this study was the potential selection and volunteering bias. More than half of eligible participants did not agree to take part. Thus, our final cohort may have selected for those more comfortable with screening for these diseases. Furthermore, this screening was offered during the first medical interview on the day of arrival at the CRA following arrest by the police. Detainees may be shocked by their arrest and fear that the results will be communicated to the police. This large proportion of participants refusing to participate could have constituted a recruitment bias, however, the characteristics of the included participants were congruent with the global population of the CRA in 2022, and thus representative of the CRA population (Supplementary Table 5). There may be a recollection bias when responding to the questionnaire. Several detainees could not remember when they last had unprotected sex, the date of their last medical contact, the date of their last blood transfusion, or the date of their last HIV, HCV and HBV screening. There also was no test for false negatives. If we had tested all participants using both serology and RDT, we might have found RDT negative but serology positive results.

Screening for Chlamydia Trachomatis, Gonococcus, tuberculosis, and syphilis was not included in this study, and it is not currently recommended to screen migrants for these infections. However, it is important to extend screening to this population to control one of the reservoirs of these infections and limit their transmission, particularly within the French population, as detainees may be released and remain on French territory. Screening for these diseases at an asymptomatic stage would allow effective treatments and reduce complications (pelvic inflammatory disease, infertility and transmission to infants and sexual partners). An American study suggested that sexually active migrants should be offered screening for sexually transmitted diseases (STDs) [36]. A Finnish study suggested that migrants were a high-risk population and supported systematic syphilis screening [32], and the recommendations in Finland are to systematically include syphilis when screening for STDs. Some countries have implemented screening programmes among newly arrived migrants, especially for tuberculosis [36, 37]. On the contrary, another American study indicated that screening for Chlamydia Trachomatis and gonococcus did not seem appropriate for routine screening of refugee women [38].

A future study could also analyse the prevalence of Chlamydia trachomatis, gonococcus, syphilis and tuberculosis infections in CRAs. In addition, it would be interesting to perform an economic analysis of the cost-benefit for the screening, considering the RDT versus serology accuracy.

## Conclusion

The objective of this study was to estimate the prevalence of HIV, HCV and HBV among detainees at the Nîmes CRA, through systematic RDT screening. The TRODUMCRA study provided prevalence data for detainees: 0% HIV, 4.64 (95% CI: [2.42; 6.86]) % HCV and 2.32 (95% CI: [1.01; 4.52]) % HBV. With the increasing rates of migration to Europe, the number of detainees in France’s CRAs is increasing every year. This study highlighted the need to screen all detainees for HIV, HCV and HBV infection, and demonstrated the acceptability of RDTs. A multicentre study with a larger sample size would allow a more precise estimate of prevalence and the differences in prevalence between CRAs depending on the associated factors. Further studies could help determine whether a targeted screening strategy would be necessary for these infections.

## Electronic Supplementary Material

Below is the link to the electronic supplementary material.

Supplementary Material 1

## Data Availability

The data that support the findings of this study are available upon request from the corresponding author.Data is provided within the manuscript or supplementary information files.

## Abbreviations

CRA: administrative detention centre
HAS: French Health Service
HBsAg: hepatitis B surface antigen
HBV: Hepatitis B
HCV: Hepatitis C
HIV: Human immunodeficiency virus
IOM: International Organization of Migration
RDT: Rapid Diagnostic Test
RNA: Ribonucleic Acid
STDs: sexually transmitted diseases
UMCRA: medical unit of administrative detention centre
USA: United State of America
WHO: World Health Organization

## Declarations

Data avaibility.

The study data are available upon reasonable request to the corresponding author.

## References

[1] International Organization for Migration. About Migration [Internet]. 2022. Disponible sur: https://www.iom.int/about-migrationhttpshttps://www.iom.int/about-migration.

[2] Santoso D, Asfia SKBM, Mello MB, Baggaley RC, Johnson CC, Chow EPF, et al. HIV prevalence ratio of international migrants compared to their native-born counterparts: a systematic review and meta-analysis. eClinicalMedicine nov. 2022;53:101661. 10.1016/j.eclinm.2022.101661 PMC9486043 36147629

[3] Seedat F, Hargreaves S, Nellums LB, Ouyang J, Brown M, Friedland JS. How effective are approaches to migrant screening for infectious diseases in Europe? A systematic review. Lancet Infect Dis Sept. 2018;18(9):e259–71. 10.1016/S1473-3099(18)30117-8 29778396

[4] European Centre for Disease Prevention and Control. Epidemiological assessment of hepatitis B and C among migrants in the EU/EEA. Stockholm: ECDC; 2016.

[5] Chappuis M, Pauti MD, Tomasino A, Fahet G, Cayla F, Corty JF. Knowledge of HIV and Hepatitis B and C status among people living in extreme poverty in France, in 2012. Médecine Mal Infect mars. 2015;45(3):72–7. 10.1016/j.medmal.2015.01.008 25660328

[6] La Cimade. Rapport 2022 sur les centres et locaux de rétention administrative. 2023.

[7] Copenhagen, WHO Regional Office for Europe. Addressing the health challenges in immigration detention, and alternatives to detention: a country implementation guide. Licence: CC BY-NC-SA 3.0 IGO; 2022.

[8] Le ministre de l’intérieur et le ministre des solidarités et de la santé. Instruction du Gouvernement du 11 février 2022 relative aux centres de rétention administrative– organisation de la prise en charge sanitaire des personnes retenues.

[9] Pétiau A. Exercice de la médecine générale au centre de rétention administrative de Rennes. [France]: Rennes 1; 2009.

[10] Panneer N, Lontok E, Branson BM, Teo CG, Dan C, Parker M, et al. HIV and Hepatitis C Virus Infection in the United States: whom and how to test. Clin Infect Dis 15 sept. 2014;59(6):875–82. 10.1093/cid/ciu396 24867787

[11] Barbot F. DEPICRA: une étude sur les facteurs influençant l’accès Au dépistage Du VIH et des hépatites B et C chez les patients dans les centres de rétention administrative français. [France]: Montpellier-Nîmes; 2022.

[12] Muñoz-Chimeno M, Valencia J, Rodriguez-Recio A, Cuevas G, Garcia-Lugo A, Manzano S, et al. HCV, HIV AND HBV rapid test diagnosis in non-clinical outreach settings can be as accurate as conventional laboratory tests. Sci Rep 9 mai. 2023;13(1):7554. 10.1038/s41598-023-33925-2 PMC10170094 37160925

[13] Hepatitis Blood Foundation. Hepatitis B Blood Tests [Internet]. 2023. Disponible sur: https://www.hepb.org/prevention-and-diagnosis/diagnosis/hbv-blood-tests/.

[14] Haute autorité de santé. Place des tests rapides d’orientation diagnostique (TROD) dans la stratégie de dépistage de l’hépatite B. juill 2016;118.

[15] Fonseca K, Di Francesco L, Galli R, Hogg B, Schechter M, Kane S et al. Results from a multi-centre Canadian clinical trial of a rapid HIV antibody test for use in Point-of-care, Clinical and Laboratory settings. The XV International AIDS Conference Abstract no. MoPeB3109. www.iasociety.org.

[16] Poiteau L, Soulier A, Lemoine M, Mohammed Z, Wlassow M, Rwegasha J, et al. Performance of a new rapid diagnostic test for the detection of antibodies to hepatitis C virus. J Virol Methods nov. 2018;261:153–5. 10.1016/j.jviromet.2018.08.019 30176305

[17] La Banque Mondiale. Prévalence du VIH, total (% de la population âgée de 15 à 49 ans)| Data [Internet]. 2021. Disponible sur: https://data.worldbank.org/indicator/SH.DYN.AIDS.ZS?end=2021&most_recent_value_desc=true&start=1990&view=chart

[18] Institut de veille sanitaire. Prévalence Des hépatite B Et C en France en 2004. Saint-Maurice; 2006. pp. d–c.

[19] Schweitzer A, Horn J, Mikolajczyk R, Krause G, Ott J. Estimations of worldwide prevalence of chronic hepatitis B virus infection: a systematic review of data published between 1965 and 2013. Lancet. 2015;386(10003):1546–55. 26231459 10.1016/S0140-6736(15)61412-X

[20] Rémy AJ. Première étude sur le dépistage et la prise en charge des hépatites virales, des addictions et du VIH en centre de rétention administrative en France. J Afr Hépato-Gastroentérologie juin. 2008;2(1–2):37–8.

[21] Bozorgmehr K, Samuilova M, Petrova-Benedict R, Girardi E, Piselli P, Kentikelenis A. Infectious disease health services for refugees and asylum seekers during a time of crisis: a scoping study of six European Union countries. Health Policy Sept. 2019;123(9):882–7. 10.1016/j.healthpol.2018.04.003 29673804

[22] Pioche P C. Estimation de la prévalence de l’hépatite C en population générale, France métropolitaine, 2011. Hépat B C Données Épidémiologiques Récent. 2016;17 mai(BEH 13–14):224–9.

[23] Coppola N, Alessio L, Gualdieri L, Pisaturo M, Sagnelli C, Caprio N et al. sept. Hepatitis B virus, hepatitis C virus and human immunodeficiency virus infection in undocumented migrants and refugees in southern Italy, January 2012 to June 2013. Eurosurveillance [Internet]. 3 2015;20(35). Disponible sur: https://www.eurosurveillance.org/content/10.2807/1560-7917.ES.2015.20.35.30009. 10.2807/1560-7917.ES.2015.20.35.30009 26530499

[24] Bil JP, Schrooders PA, Prins M, Kouw PM, Klomp JH, Scholing M et al. Integrating hepatitis B, hepatitis C and HIV screening into tuberculosis entry screening for migrants in the Netherlands, 2013 to 2015. Eurosurveillance [Internet]. 15 mars 2018;23(11). Disponible sur: https://www.eurosurveillance.org/content/10.2807/1560-7917.ES.2018.23.11.17-00491. 10.2807/1560-7917.ES.2018.23.11.17-00491 PMC5861593 29560855

[25] Prestileo T, Di Marco V, Dino O, Sanfilippo A, Tutone M, Milesi M, et al. Effectiveness of a screening program for HBV, HCV, and HIV infections in African migrants to Sicily. Dig Liver Dis juin. 2022;54(6):800–4. 10.1016/j.dld.2021.08.024 34649829

[26] Vignier N, Moussaoui S, Marsaudon A, Wittwer J, Jusot F, Dourgnon P. Burden of infectious diseases among undocumented migrants in France: results of the Premiers Pas survey. Front Public Health 4 août. 2022;10:934050. 10.3389/fpubh.2022.934050 PMC9386354 35991026

[27] Duracinsky M, Yaya I, Yombo-Kokule L, Thonon F, Rousset-Torrente O, Roudot-Thoraval F et al. Étude de la prévalence de l’infection à VIH et des hépatites B et C, et chez les migrants réguliers en France: données de l’étude STRADA (2017–2020). Médecine Mal Infect Form. juin 2022;Volume 1(Issue 2, supplement):Page S27.

[28] Cortesi PA, Fornari C, Conti S, Antonazzo IC, Ferrara P, Ahmed A, et al. Hepatitis B and C in Europe: an update from the global burden of Disease Study 2019. Lancet Public Health Sept. 2023;8(9):e701–16. 10.1016/S2468-2667(23)00149-4 PMC11138131 37633679

[29] Davlidova S, Haley-Johnson Z, Nyhan K, Farooq A, Vermund SH, Ali S. Prevalence of HIV, HCV and HBV in Central Asia and the Caucasus: a systematic review. Int J Infect Dis mars. 2021;104:510–25. 10.1016/j.ijid.2020.12.068 PMC11094609 33385583

[30] Lombardi A, Mondelli MU, ESCMID Study Group for Viral Hepatitis (ESGVH). Hepatitis C: Is eradication possible? Liver Int. mars,. 2019;39(3):416–26. 10.1111/liv.14011 30472772

[31] Bonneton M. Prévalence et facteurs de risque d’infections sexuellement transmissibles dans la population de migrants consultant Au Centre gratuit d’information de dépistage et de diagnostic d’infection par les virus de l’immunodéficience humaine, des hépatites virales et des infections sexuellement transmissibles. [France]: Paris Est Créteil; 2019.

[32] Tiittala P, Ristola M, Liitsola K, Ollgren J, Koponen P, Surcel HM, et al. Missed hepatitis b/c or syphilis diagnosis among kurdish, Russian, and Somali origin migrants in Finland: linking a population-based survey to the national infectious disease register. BMC Infect Dis déc. 2018;18(1):137. 10.1186/s12879-018-3041-9 PMC5859750 29558910

[33] Dalekos GN, Zervou E, Karabini F, Tsianos EV. Prevalence of viral markers among refugees from southern Albania: increased incidence of incidence with hepatitis A, B and D viruses. Eur J Gastroenterol Hepatol juin 1995;553–8. 7552639

[34] Chemaitelly H, Mahmud S, Rahmani AM, Abu-Raddad LJ. The epidemiology of hepatitis C virus in Afghanistan: systematic review and meta-analysis. Int J Infect Dis Nov. 2015;40:54–63. 10.1016/j.ijid.2015.09.011 26417880

[35] Richter C, Ter Beest G, Gisolf EH, Van Bentum P, Waegemaekers C, Swanink C, et al. Screening for chronic hepatitis B and C in migrants from Afghanistan, Iran, Iraq, the former Soviet Republics, and Vietnam in the Arnhem region, the Netherlands. Epidemiol Infect oct. 2014;142(10):2140–6. 10.1017/S0950268813003415 PMC9151278 24398373

[36] Adachi M, Takemura S. Outcomes of systemic screening for syphilis, gonorrhoea and Chlamydia trachomatis among immigrant visa applicants migrating from Japan to the US in 2016–2019. J Travel Med. 2022;25(juill):taac084. 10.1093/jtm/taac084 35880852

[37] Pareek M, Baussano I, Abubakar I, Dye C, Lalvani A. Evaluation of immigrant tuberculosis screening in industrialized countries. Emerg Infect Dis Sept. 2012;18(9):1422–9. 10.3201/eid1809.120128 PMC3437731 22931959

[38] Kaiser R, Stauffer WM, Painter J, Weinberg M, Berman S, Mamo B. Sexually transmitted infections in newly arrived refugees: is routine screening for Neisseria gonorrheae and Chlamydia trachomatis infection indicated? Am J Trop Med Hyg 1 févr. 2012;86(2):292–5. 10.4269/ajtmh.2012.11-0527 PMC3269283 22302865

