# Supplementary material for: Prevalence of HIV, Hepatitis C and Hepatitis B Infection Among Detainees in a French Administrative Detention Centre
Source: J Epidemiol Glob Health. 2024 May 13;14(3):923–32. doi: 10.1007/s44197-024-00238-0 (PMC11442733; doi:10.1007/s44197-024-00238-0)
Supplement: Supplementary file 1 — Supplementary Material 1 [file 44197_2024_238_MOESM1_ESM.docx]

# Supplementary information

**Supplementary Table 1**: patient report form

| **TRODUMUCRA e-CRF** | |
| --- | --- |
| **Inclusion** | |
| Inclusion no. |  |
| Inclusion date |  |
| Gender | ☐ Male ☐ Female  ☐ Other (please specify): |
| Year of birth |  |
| Month of birth |  |
| Age at inclusion |  |
| Country of origin |  |
| Do you speak French? | ☐ Yes ☐ No |
| - If not, what language: |  |
| **Inclusion criteria** | |
| Has the person given free, informed, express oral consent? | ☐ Yes ☐ No |
| Person detained at the administrative detention centre in Nîmes? | ☐ Yes ☐ No |
| Is the person an adult (18 years or older)? | ☐ Yes ☐ No |
| **Non-inclusion criteria** | |
| Person for whom it is impossible to give clear information? | ☐ Yes ☐ No |
| **TRODUMCRA questionnaire**  **Patient characteristics** | |
| 1. Are you: | ☐Single ☐ Cohabiting  ☐ Married ☐ Separated |
| 1. When did you arrive in France? *(year)* |  |
| 1. Have you ever seen a doctor since your arrival in France? | ☐ Yes ☐ No |
| 1. If so, what was the date of your last medical contact? *(month, year)* |  |
| 1. Have you ever been screened for HIV, hepatitis C and hepatitis B ? | ☐ Yes ☐ No |
| - If so, when? *(month, year)*   *(If the month is unknown, write 06)* |  |
| - If so, what was the result of your HIV test? | ☐ Positive ☐ Negative ☐ Not done ☐ Don’t know |
| - If so, what was the result of your test for hepatitis C? | ☐ Positive ☐ Negative ☐ Not done ☐ Cured ☐ Don’t know |
| - If so, what was the result of your test for hepatitis B? | ☐ Positive ☐ Negative ☐ Not done ☐ Don’t know |
| **Associated factors** | |
| 1. Are you aware of any HIV, hepatitis C or hepatitis B infection? | ☐ Yes ☐ No |
| 1. Have you ever undergone transfusion? | ☐ Yes ☐ No |
| - If so, in what country? |  |
| - If so, when? *(year)* |  |
| 1. Have you ever taken intravenous drugs? | ☐ Yes ☐ No |
| 1. Have you ever taken drugs nasally? | ☐ Yes ☐ No |
| - If the answer to question 3 and/or 4 is yes, have you ever shared your equipment? | ☐ Yes ☐ No |
| 1. Have you ever had unprotected sex? | ☐ Yes ☐ No |
| - If so, when was the last time? *(year)* |  |
| 1. Have you got any body-piercing? | ☐ Yes ☐ No |
| - If so, how was it done? | ☐ Professional ☐ Artisan |
| 1. Have you been tattooed? | ☐ Yes ☐ No |
| - If so, how was it done? | ☐ Professional ☐ Artisan |

**Supplementary Table 2:** “other” reasons for study exclusion

| **Other reason (n=29)** |
| --- |
| - Detainees released by the judge during the reflection period (n=23) - Detainees refused to come to UMCRA (n=2) - Other (n=4) - 2 transfers (1 to prison and 1 to neighbouring CRA) - 1 HIV seropositivity already known - 1 STDs test performed recently in prison without risky behaviour |

**Supplementary Table 3:** countries of origin with fewer than 2% participants

| **Characteristics** | **Population (n=345)** |
| --- | --- |
| ***Other country of origin**** | ***65 (19%)*** |
| Libya | 5 (1%) |
| Serbia | 5 (1%) |
| Pakistan | 4 (1%) |
| Senegal | 4 (1%) |
| Afghanistan | 3 (1%) |
| Armenia | 3 (1%) |
| Columbia | 3 (1%) |
| Union of the Comoros | 3 (1%) |
| Italy | 3 (1%) |
| Republic of the Congo | 2 (1%) |
| Gambia | 2 (1%) |
| Ivory Coast | 2 (1%) |
| Mali | 2 (1%) |
| Poland | 2 (1%) |
| Slovakia | 2 (1%) |

*for origins n=1, countries are: Brazil, Burundi, Cameroon, Cape Verde, Croatia, Cuba, Dominican Republic, Gabon, Ghana, Mauritius, Moldova, Philippines, Portugal, Russia, Saint-Martin, Somalia, Sudan, Togo, Egypt, United Kingdom

**Supplementary Information 4.** Patient information letter (English version), available in 17 other languages

“TRODUMCRA” Study

**Assessment of the prevalence of HIV, Hepatitis C and Hepatitis B infections in detainees at the Nîmes Administrative Detention Center (CRA)**

Dear Sir, Madam,

The physician at the Administrative Detention Center’s Medical Unit is offering you the opportunity to participate in a study under the framework of a public-interest mission that the Nîmes CHU is promoting. Before making a decision, it is important that you carefully read over these pages, which will provide you with the necessary information regarding the different aspects of this study. Do not hesitate to ask any questions that you deem useful to the physician.

Your participation is completely voluntary. If you would like to take part in this study, you will continue to benefit from the best possible medical care, in accordance with existing knowledge. Your verbal consent will attest to your final agreement to participate in the study.

**Presentation of the study**

This study’s objective is to estimate the prevalence of HIV, Hepatitis C and the active form of Hepatitis B in detainees at the Nîmes Administrative Detention Center. Their prevalence designates the overall health of a population at any given moment. This study attempts to find the number of people affected by HIV, Hepatitis C and the active form of Hepatitis B compared to the total number of people detained at the center.

We would like to improve detainees’ care at the Administrative Detention Center (CRA) by offering everyone with systematic screenings for HIV, Hepatitis C and B.

**Sequence of events**

Normally, people do not necessarily get screened for HIV, Hepatitis C & B when they visit the detention center’s medical unit. If you accept to participate in this study, you will be able to benefit from a rapid screening (the results of the screening for these diseases will be known within less than 30 minutes). For this screening, the nurse will take several droplets of blood from your finger. The blood will then be tested and will allow you to find out whether or not you are positive or negative for any of these diseases. These are called Rapid Diagnostic Tests (TROD).

For the study’s purposes, the nurse will also ask you several questions (specifically regarding your personal life, sex life and any eventual drug use) to determine if there are factors associated with these diseases.

The results will be communicated to you within 30 minutes of taking the rapid test with the nurse at the medical unit.

If one of the rapid tests is positive, you will be seen in consultation with the physician who will explain the results to you, follow-up treatment, answer your questions and let you know how you can prevent any risks. A blood test for serological purposes will be conducted afterwards to confirm the results of this test.

If the rapid tests are negative, the nurse will then inform you on how to prevent the risk of HIV and Hepatitis B&C.

If you refuse to participate in the study, you may – if you wish – still benefit from this rapid screening for HIV and Hepatitis B & C.

**Study population**

We offer everyone at the detention center the opportunity to participate in the study (except for those who arrive on weekends or holidays when there are no physicians present to include the patient in the study and these new arrivals have already departed from the center by the following business day).

**Expected benefits for participants in the study**

HIV, Hepatitis B & C may infect people without necessarily presenting symptoms. These people are called asymptomatic, which means that they are sick, but do not know it most of the time because they have no symptoms. By getting screened, you benefit from a free HIV and Hepatitis B & C screening and in case of positive results, you will be monitored and receive helpful advice to avoid complications related to the delayed discovery of these diseases.

In addition, this screening makes it possible to decrease the spread of these diseases throughout the entire community.

**Potential risk(s)**

The risks incurred by the study are minimal. However, an infection may occur in the area where your blood was sampled and some people might feel dizzy. The amount of time it takes for the blood to coagulate may also take longer for a hemophiliac.

**What are your rights?**

Your physician must provide you with all necessary explanations regarding this study. If you would like to withdraw at any time and for any reason, you will continue to benefit from medical follow-ups and this will not affect your monitoring in the future.

Under the framework of the study, your personal data will be digitally processed so we can analyze the results of the study in terms of the objectives that were presented to you. The Nîmes CHU is the data processing manager. The study’s physician and other personnel will collect information regarding your health, participation in the study and, if applicable, your living habits as well as, inasmuch as this data is necessary for the study, data related to your sex life. This information, called “personal information,” is noted on forms, called observational notebooks, provided by the study’s promoter. Only the information strictly necessary for processing and the purposes of the study will be collected. This data will be kept for up to two years after the last publication of the research results or, in case there is no publication, until the final research report has been signed. They will then be archived either on paper or digitally for a period of 15 years after the study has ended in accordance with the regulations in effect. To ensure the confidentiality of your personal information, neither your name nor any other directly identifiable information will be filled out in the observational notebooks or in any other file or sample that the study’s physician will provide to the promoter or the representatives authorized by the promoter. You will only be identified by a code and your initials. The code is used so that the study’s physician can identify you if necessary.

The legal basis for the processing of your data is a public-interest mission. In accordance with the provisions from the law related to data privacy (law #78-17 from January 6, 1978 related to data privacy modified by law #2018-493 from June 20, 2018 related to the protection of personal data) and the general data protection regulations (EU regulation 2016/679), you have the following rights regarding the data that we collect under the framework of this study:

• the right to request information regarding the processing of your data.

• the right to request that your data be corrected if it is incorrect or incomplete. While we examine your request, you have the right to limit the processing of your data.

• the right to request that your data be transferred to you or someone else in a regularly used format.

• the right to withdraw your consent or oppose the processing of your personal data, at any time, without having to justify your decision. No other data will then be collected after your consent is withdrawn.

• If you withdraw your consent or if you oppose the processing of your data, you can request that the data already collected be erased if there does not exist any legal requirement that necessitates their use. However, please note that any data, which would already have been processed with your initial consent, will be kept so as not to hinder or compromise the objectives of the study (Articles 17.3.C & 17.3.D of the GDPR).

You can exercise these rights by requesting them in writing with the study’s physician. The promoter will respond to your requests inasmuch as possible in accordance with their other legal and regulatory obligations and when the law requires it. If you believe that your rights are not respected, you can submit a claim to the CNIL electronically (<https://www.cnil.fr/fr/plaintes>) or by mail (CNIL – 3 Place de Fontenoy – TSA 80715 – 75334 PARIS CEDEX 07).

You also have a right to oppose the communication of confidential information likely to be used under the framework of this study and be processed. You can also access, directly or through the physician of your choice, all of your medical data in application of the provisions from article L1111-7 of the Public Health Code. These rights are exercised with your treating physician, who already knows your identity, under the framework of the study.

The competent authorities and the promoter or their authorized representatives will also be able to access your medical archives and your research file, in order to verify the data collected under the framework of the study.

Your personal encrypted information may be used for other scientific research on your disease or other diseases, always in accordance with the applicable laws and regulations.

If you have other questions regarding the collection and use of your personal information or your rights related to this information, please contact the Data Protection Officer at Nîmes CHU ([*dpd@chu-nimes.fr*](mailto:dpd@chu-nimes.fr)) or the study’s physician.

The aforementioned rights may be exercised by mail accompanied by proof of ID to the CHU DE NIMES – Place Robert Debré – Bâtiment polyvalent, Service SIH, à l’attention du Délégué à la protection des données (DPD) – 30900 NIMES or to DPD@chu-nimes.fr. For more information, you can consult the Nîmes CHU personal data protection policy on our website: <http://www.chu-nimes.fr/le-chu-de-nimes/mentions-legales.html>

If, despite the measures implemented by the promoter, you believe that your rights are not respected, you can submit a claim with the competent supervisory authority for data protection in your country of residence (CNIL for France).

In accordance with law #2012-300 from March 5, 2012 related to research involving humans:

- this research received a favorable opinion from the Institutional Review board of [name] and was declared with the National Agency for Medication and Healthcare Product Safety (ANSM),

- Nîmes CHU took out a civil liability insurance policy with HDI GLOBAL SE (#0101242214029) (Tour Opus 12, La Défense 9, 77 Esplanade du Général de Gaulle – 92914 Paris La Défense Cedex),

- people having suffered from any injury after participating in the study can exercise their rights with the regional commissions for reconciliation and compensation for medical accidents,

- when this study is over, your physician will personally inform you of the overall results as soon as they are available, if you so desire.

After having read this informational notice (with the help of a translator, if necessary), do not hesitate to ask the physician any questions that you would like. After a period of consideration, if you accept to participate in this study, you must fill out and sign the consent form for participation. A copy of the entire document will be given to you.

In addition, all the information that will be collected for the study, i.e. all of the interviews carried out at the Administrative Detention Center’s Medical Unit as well as the exam requests and results will be covered by medical confidentiality and will never be revealed to third parties.

*We thank you for your attention.*

**If you have any questions, do not hesitate to ask your physician.**

| **Name and contact information**  **of the chief investigator** |
| --- |
| Dr. Mélanie KINNE  Administrative Detention Center Medical Unit (UMCRA)  Nîmes CHU  Tel.: 04.66.20.45.71 |

**We thank you, in advance, Madam or Sir, for the assistance you are contributing to medical research.**

**Supplementary Table 5:** comparison of the analysed population against global population of the centre

| **Characteristics** | **Population TRODUMCRA (n=345)** | **Population CRA Nîmes in 2022 (n= 844)*** |
| --- | --- | --- |
| ***Sex,*** male | 309 (90%) | 768 (91%) |
| ***Country of origin*** |  |  |
| Algeria | 119 (34%) | 290 (34.4%) |
| Tunisia | 43 (12%) | 96 (11.4%) |
| Morocco | 37 (11%) | 97 (11.5%) |
| Georgia | 23 (7%) | 37 (4.4%) |
| Guinea | 15 (4%) | 26 (3.1%) |
| Romania | 14 (4%) | 37 (4.4%) |
| Albania | 8 (2%) | 24 (2.8%) |
| Turkey | 6 (2%) | 22 (2.6%) |
| Nigeria | 9 (3%) | 20 (2.4%) |
| Afghanistan | 3 (1%) | 1. (2%) |

*Data taken from "La Cimade. Rapport 2022 sur les centres et locaux de rétention administrative. 2023”
